# Psychological Distress among Italian University Students Compared to General Workers during the COVID-19 Pandemic

**DOI:** 10.3390/ijerph18052503

**Published:** 2021-03-03

**Authors:** Annunziata Romeo, Agata Benfante, Lorys Castelli, Marialaura Di Tella

**Affiliations:** Department of Psychology, University of Turin, Via Verdi 10, 10124 Turin, Italy; annunziata.romeo@unito.it (A.R.); lorys.castelli@unito.it (L.C.); marialaura.ditella@unito.it (M.D.T.)

**Keywords:** COVID-19 pandemic, mental health, anxiety symptoms, depressive symptoms, university students, workers

## Abstract

The COVID-19 pandemic induced numerous changes in the daily life of every individual, with important social, economic, and psychological consequences. Particularly, the psychological impact encountered among students might be affected by social isolation, concern for personal health and for the health of family members and friends, and uncertainty about academic progress. The present study aimed to investigate the psychological impact of the COVID-19 outbreak on Italian university students compared to general workers. The responses of 956 participants (478 university students and 478 workers) were included in the final dataset. Participants were asked to provide sociodemographic and occupation-related information, and to complete: (1) COVID-19-related questions; (2) health-related visual analogue scales; (3) State-Trait Anxiety Inventory-Form Y1 (STAI Y1); and (4) the Beck Depression Inventory (BDI-II). Results of comparisons between university students and general workers revealed that the former reported higher levels of anxiety and depressive symptoms. Furthermore, regression analyses showed that in university students, gender, health evaluation, and health concern and gender, educational level, and health evaluation significantly predicted anxiety and depressive symptoms, respectively. Taken together these findings suggest that specific factors could predispose University students to a high risk of developing mental health symptoms as a consequence of the COVID-19 pandemic.

## 1. Introduction

The COVID-19 pandemic induced numerous changes in daily life of every individual as a result of the measures to contain the infection, with important social, economic, and psychological consequences.

In Italy, as in the rest of the world, high levels of psychological distress have been found in both the general population and specific categories of workers, such as healthcare workers [1,2,3]. Several studies have also focused on the prevalence of psychological distress in young people who have experienced drastic change in their daily habits, as a result of the closure of schools and universities and the transition to online teaching [4,5,6,7,8,9,10]. Previous studies have found that university students are at high risk of developing mental health problems, such as anxiety and depression [11,12,13]. Therefore, this category might be even more at risk of experiencing high levels of psychological distress as a result of the COVID-19 outbreak. The reduction of social interactions due to the measures introduced to contain the spread of contagion, the concern for personal health and for the health of family members and friends, the uncertainty about the future and academic progress, but also the financial and work-related worries might affect the psychological well-being and the mental health of university students [5,6,14,15].

The main aim of the present study was to investigate the psychological impact of the COVID-19 outbreak on Italian university students. Firstly, we compared the levels of anxiety and depression symptoms between university students and general workers. Secondly, we examined sociodemographic factors and other characteristics that could significantly predict psychological distress in university student and general worker samples, separately considered, during the lockdown in Italy. Exploring the levels of psychological distress among university students may allow clinicians to gain insights into baseline levels of mental health as a result of the pandemic.

## 2. Materials and Methods

The data were collected using an online survey from 19 March to 5 April 2020. An anonymized, individual, and unique code to complete the survey was provided to each of those who agreed to participate in the study. A snowball sampling strategy was employed, wherein the participants were initially recruited via online advertisements and were encouraged to pass the survey link to others. The responses of 956 participants (478 university students—B.Sc., M.Sc., Ph.D. students—and 478 general workers) were included in the final dataset. The original dataset was composed of 1321 participants, whose characteristics and analyses were reported in a previous study [1].

Participants were asked to provide sociodemographic and occupation-related information (age, gender, profession, marital status, educational level, and study satisfaction for students only). Moreover, participants were asked to complete: (1) COVID-19-related questions, (2) health-related visual analogue scales (VASs); (3) State-Trait Anxiety Inventory-Form Y1 (STAI Y1) to evaluate the presence of anxiety symptoms; and (4) the Beck Depression Inventory (BDI-II) to assess the levels of depressive symptoms.

In order to explore the psychological impact of the COVID-19 outbreak on our group of university students, descriptive analyses and chi-square tests were first performed on sociodemographic variables (i.e., marital status, educational level, and study satisfaction) and COVID-19-related questions. Secondly, independent *t*-tests were run to evaluate the presence of possible differences between university students and general workers in age, health-related questions, and psychological variables (anxiety/depressive symptoms). The effect size was determined by calculating Cohen’s *d*.

Finally, four hierarchical multiple regression analyses were run to assess whether sociodemographic variables, health-related items, and COVID-19-related questions were significant predictors of the psychological outcomes in the samples of university students and of general workers, separately considered. STAI Y1 and BDI-II scores were used as dependent variables. In all regression models, independent variables were entered as follows: sociodemographic variables in the first block, health-related items in the second block, and COVID-19-related questions in the third block. The enter method was used to include the variables of the predictor groups. Collinearity was assessed through the statistical factor of tolerance and variance inflation factor (VIF).

All the statistical analyses were conducted using Statistical Package for the Social Sciences, version 26.0 (IBM SPSS Statistics for Windows, Armonk, NY, USA: IBM Corp.).

The study was approved by the University of Turin Ethics Committee (Protocol No. 142069) and conducted according to the Declaration of Helsinki. All the participants gave their written informed consent to participate in the study.

## 3. Results

Sociodemographic characteristics, COVID-19-related information, and psychological data (i.e., health-related items, BDI, and STAI Y1) for the student and worker groups are presented in Table 1.

With regard to sociodemographic characteristics, the majority of our university students had a B.Sc. or a M.Sc. degree (57%), were not in a relationship (96%), and considered themselves moderately (48%, 229) satisfied with their studies (“quite a bit”: 28%, 134; “a little bit”: 19%, 92; “not at all”: 4%, 19). Concerning COVID-19-related questions, 4% of our students reported having had contact with other people positive for COVID-19, while 46% and 12% of them reported that they knew about others who were positive for COVID-19 or who died of COVID-19, respectively.

The results of comparisons between student and worker participants on psychological variables revealed that university students reported higher levels of both anxiety (*p* = 0.013, *d* = 0.16) and depressive (*p* < 0.001, *d* = 0.28) symptoms compared to general workers (Table 1).

Four hierarchical multiple regression analyses were further performed to assess whether sociodemographic variables (age, gender, marital status, educational level), health-related items (health evaluation—”How do you currently rate your health?”—and health concern—”How concerned are you about contracting COVID-19?”), and COVID-19-related questions (knowing of others who tested positive for COVID-19, knowing of others who died of COVID-19, having had contact with others who tested positive for COVID-19) significantly predicted STAI Y1 and BDI scores in the university student group and in the general worker sample, separately considered.

With regard to anxiety symptom scores in the university student group, the full regression model statistically significantly predicted the STAI Y1 total score, F(9, 468) = 17.740, *p* < 0.001, adjusted R^2^ = 0.24. Among all the predictors, gender (*β* = −0.149, *p* < 0.001), VAS health evaluation (*β* = −0.268, *p* < 0.001), and VAS health concern (*β* = 0.346, *p* < 0.001) were statistically significant (Table 2). Particularly, being female, rating lower one’s own health, and being more concerned about contracting COVID-19 were found to be associated with higher levels of anxiety symptoms.

Regarding anxiety symptom scores in the general worker sample, the full regression model statistically significantly predicted the STAI Y1 total score, F(9, 468) = 17.740, *p* < 0.001, adjusted R^2^ = 0.24. Among all the predictors, gender (*β* = −0.149, *p* < 0.001), VAS health evaluation (*β* = −0.268, *p* < 0.001), and VAS health concern (*β* = 0.346, *p* < 0.001) were statistically significant (Table S1). Particularly, being female, rating lower one’s own health, and being more concerned about contracting COVID-19 were found to be associated with higher levels of anxiety symptoms.

As far as depressive symptoms are concerned, the full regression model statistically significantly predicted the BDI total score, F(9, 467) = 9.859, *p* < 0.001, adjusted R^2^ = 0.14 in the university student group. Among all the predictors, gender (*β* = −0.112, *p* = 0.011), educational level (*β* = −0.123, *p* = 0.010), and VAS health evaluation (*β* = −0.342, *p* < 0.001) were statistically significant (Table 3). Particularly, being female, having a lower educational level (high school diploma), and rating lower one’s own health were found to be associated with higher levels of depressive symptoms.

Regarding depressive symptom scores in the general worker sample, the full regression model statistically significantly predicted the BDI total score, F(9, 467) = 9.859, *p* < 0.001, adjusted R^2^ = 0.14. Among all the predictors, gender (*β* = −0.112, *p* = 0.011), educational level (*β* = −0.123, *p* = 0.010), and VAS health evaluation (*β* = −0.342, *p* < 0.001) were statistically significant (Table S2). Particularly, being female, having a lower educational level, and rating lower one’s own health were found to be associated with higher levels of depressive symptoms.

In all regression analyses, the statistical factor of tolerance and VIF showed that there were no interfering interactions between the variables.

## 4. Discussion

The main aim of the present study was to investigate the psychological impact of the COVID-19 pandemic on Italian university students. In order to reach this goal, we first compared university students with different kinds of workers for levels of anxiety and depressive symptoms, and secondly, we examined the possible significant predictors of psychological distress (i.e., anxiety/depressive symptoms) in the group of university students and in the sample of general workers, separately considered.

As far as the first goal is concerned, results showed that university students reported higher levels of both anxiety and depressive symptoms than general workers. Indeed, 75% and 38% of university students scored above the cutoff point for anxiety and depression, respectively. In line with our results, the study of Odriozola-Gonzalez et al. showed that students experienced significantly higher depression, anxiety, and stress scores compared to the different groups of employees [9].

These findings are not surprising considering that adults tend to use more adaptive strategies than young people, and consequently they are less at risk to develop psychological distress during a stressful event [16]. Moreover, these results could be explained by the shift to distance education. Indeed, students’ anxiety has been associated with the effect of COVID-19 on their studies [17] and with their uncertain future employment [18]. Still, the quarantine caused significant distance between people, and it is likely that the absence of interpersonal relationships could be associated with increased psychological distress. Previous studies suggested that anxiety disorders are more likely to occur in the absence of interpersonal communication [19,20]. Universities play a significant role in satisfying health, education, and safety needs of students, and so the actions taken to contain the pandemic have inevitably affected students’ mental health and well-being [15].

With regard to the second aim of the present study, we investigated which factors could significantly predict the high levels of anxiety and depressive symptoms displayed by our university students and general workers, separately considered. In the university student group, significant predictors of those symptoms were found to be being female, rating lower one’s own health, and being more concerned about contracting COVID-19 for anxiety symptoms and being female, having a lower educational level, and rating lower one’s own health for depressive symptoms. Similarly, in the general worker sample, significant predictors were found to be being female, rating lower one’s own health, and being more concerned about contracting COVID-19 for anxiety symptoms and being female, having a lower educational level, and rating lower one’s own health for depressive symptoms.

As far as gender differences are concerned, it is widely known that women are at higher risk of anxiety and depression when compared to men. Therefore, our results are in line with the majority of previous studies, which showed higher levels of psychological distress in females than males in student samples [5,8]. The only exception seems to be represented by the study of Cao et al., which found similar anxiety levels in male and female students as a result of the pandemic [4].

Similarly, regarding education level, previous studies showed that first year students reported more mental health symptoms than others [9]. More generally, a high educational level seems to represent a protective factor against the development of psychological distress in adult individuals [21].

Unexpectedly, in both university student and general worker samples, having had contact with others who tested positive for COVID-19 seemed not to represent an influencing factor to develop major risk of psychological distress. Likewise, knowing of others who are positive does not appear to represent a risk factor to depressive and/or anxiety symptoms. These findings are in contrast with those of previous studies, which found a significantly higher risk of emotional and anxiety disorders among students who had relatives or friends positive for COVID-19 [4,6] or had contact with others who tested positive for COVID-19 [14]. Moreover, the study of Chi et al. found that knowing people who had been isolated during the COVID-19 pandemic was significantly associated with higher levels of anxiety, depression, and Post Traumatic Stress Disorder [22].

Conversely, the results of our study showed that having concern about contracting COVID-19 (VAS health concern) appears to predispose particularly to high levels of anxiety symptoms, while rating low one’s own health (VAS health evaluation) seems to be associated with a higher risk of developing both anxiety and depressive symptoms in our groups of participants. In line with our findings, the study of Li et al., which investigated the levels of psychological distress before and after 2 weeks of confinement, revealed that the fear of being infected by COVID-19 had a significant association with reduced positive effect, while the belief regarding how many people were infected or died by COVID-19 was associated with an increase in anxiety and depression [23]. Finally, the study of Elmer et al. found that not being worried about one’s own health but being worried about family and friends was associated with worsening of mental health [5].

Taken together these different findings suggest that students react to the pandemic with major levels of psychological distress and that this may depend on more intrinsic factors concerning, for example, worries and feelings of vulnerability, and consequently their resources to cope with them. A high tendency to worry before the pandemic outbreak has been associated with major fear about one’s own mental health [24], while lower levels of resilience have been found to be significantly related to higher levels of anxiety and depression [22]. The negative relationship between predisposition to worry and the capacity to cope with stressful events is widely documented in literature [25]. Conversely, the level of health engagement could represent a protective factor for students’ mental health [7].

The present study has also some limitations that should be considered. First, we adopted a cross-sectional study design, which does not permit us to draw firm conclusions about the causality of the emergent relationships. Future longitudinal studies should be carried out to determine the developmental trajectory and the possible predictors of mental health symptoms during the COVID-19 pandemic. Secondly, due to the use of an online survey, only self-report instruments could be administered to evaluate the presence of anxiety/depressive symptoms. Structured interviews could be employed in addition to traditional self-report measures, in order to gain a more accurate assessment of mental health symptoms. Finally, our samples of university students and general workers were not matched for age. However, it should be noted that workers are generally older than students, considering the different occupational status they have. Moreover, the general workers we recruited for the present study also included a great number of participants who have already gained a degree, and this might have contributed to the age gap that we found between these two groups.

## 5. Conclusions

The findings reported in the current study highlight the presence of higher levels of anxiety/depressive symptoms in university students compared to general workers. Furthermore, our results show that specific sociodemographic and health-related factors seem to contribute to the increased psychological distress displayed by our group of university students. 

During the extraordinary events associated with the COVID-19 pandemic, it is essential to take on students’ needs and identify signs of psychological distress or risky behaviors. Our findings provide an overview on the early effects of the pandemic on students’ mental health, and these data can be a useful litmus test for clinicians dealing primarily with young adults.

Previous studies underlined the effectiveness of online support interventions, which have been carried out both to help students with problems inherent to their academic path (motivation and reorganization of the study) and to take care of their psychological, emotional, and relational needs related to the COVID-19 pandemic [15,25,26]. Specific interventions should thus be employed to manage the negative effects of the COVID-19 outbreak on the psychological well-being of university students.

## Figures and Tables

**Table 1:** Sociodemographic characteristics of student and worker groups. Mean (SD), percentage, *t*-test, and Cohen’s *d* are listed.

	Students (*N* = 478)	Workers (*N* = 478)	Test (df)	*p*	Effect Size
Age (years)	23.4 (2.7)	33.3 (6.9)	*t*(617.21) = −29.13	<0.001	*d* = 2.07
Gender			*χ*^2^(1) = 11.13	0.001	
Male	108 (22.6%)	154 (32.2%)			
Female	370 (77.4%)	324 (67.8%)			
Educational level			*χ*^2^(1) = 37.04	<0.001	
Primary/secondary/high school diploma	206 (43.1%)	117 (24.5%)			
B.Sc. or M.Sc. degree/postgraduate qualification	272 (56.9%)	361 (75.5%)			
Marital status			*χ*^2^(1) = 191.29	<0.001	
Not in a relationship	458 (95.8%)	278 (58.2%)			
In a relationship	20 (4.2%)	200 (41.8%)			
COVID-19 questions					
Knowing of others who are positive (yes response)	219 (45.8%)	267 (55.9%)	*χ*^2^(1) = 9.64	0.002	
Knowing of others who died (yes response)	59 (12.3%)	88 (18.4%)	*χ*^2^(1) = 6.76	0.009	
Having had contact with others who tested positive (yes response)	19 (4.0%)	94 (19.7%)	*χ*^2^(1) = 56.45	<0.001	
Psychological aspects					
Health evaluation (VAS) *	8.15 (1.5)	8.2 (1.4)	*t*(954) = −0.11	0.910	*d* = 0.01
Health concern (VAS) ^#^	5.4 (2.3)	6.0 (2.3)	*t*(954) = −3.83	<0.001	*d* = 0.25
STAI Y1	49.8 (12.0)	47.8 (13.0)	*t*(948.19) = 2.48	0.013	*d* = 0.16
Scored above the STAI Y1 cutoff point (≥41)	359 (75.1%)	311 (65.1%)	*χ*^2^(1) = 11.50	<0.001	
BDI-II	12.5 (9.2)	10.0 (8.8)	*t*(953) = 4.40	<0.001	*d* = 0.28
Scored above the BDI-II cutoff point (>13)	180 (37.7%)	137 (25.9%)	*χ*^2^(1) = 8.87	0.003	

SD = standard deviation; VAS = visual analogue scale; BDI-II = Beck Depression Inventory; STAI Y1 = State-Trait Anxiety Inventory Form Y1. * Health evaluation question = “How do you currently rate your health?”. ^#^ Health concern question = “How concerned are you about contracting COVID-19?”.

**Table 2:** Hierarchical multiple regression predicting STAI Y1 scores from sociodemographic variables, health-related items, and COVID-19-related questions in the student group (*N* = 478).

STAI Y1								
Predictors	B	*β*	*t*	95% CI	Adj R^2^	F	∆R^2^	∆F
**Model 1**					0.059	8.420 **	0.066	8.420 **
Age	0.010	0.002	0.044	−0.431; 0.451				
Gender	−7.001	−0.244	−5.421 **	−9.539; −4.463				
Marital status	2.571	0.043	0.948	−2.761; 7.904				
Educational level	−2.568	−0.106	−2.129 *	−4.938 −0.198				
**Model 2**					0.242	26.377 **	0.185	58.215 **
Age	−0.158	−0.035	0.436	−0.557; 0.241				
Gender	−4.412	−0.153	−3.720 **	−6.742; −2.081				
Marital status	−3.028	0.050	1.242	−1.763; 7.819				
Educational level	−2.009	−0.083	−1.853	−4.140; 0.122				
Health evaluation	−2.273	−0.273	−6.763 **	−2.933; −1.612				
Health concern	1.766	0.341	8.382 **	1.352; 2.180				
**Model 3**					0.240	17.740 **	0.003	0.601
Age	−0.166	−0.037	−0.817	−0.565; 0.233				
Gender	−4.286	−0.149	−3.592 **	−6.630; −1.941				
Marital status	3.200	0.053	1.309	−1.604; 8.004				
Educational level	−2.035	−0.084	−1.873	−4.169; 0.100				
Health evaluation	−2.231	−0.268	−6.589 **	−2.897; −1.566				
Health concern	1.792	0.346	8.417 **	1.373; 2.210				
COVID-19_1	−0.291	−0.012	−0.273	−2.386; 1.804				
COVID-19_2	0.045	0.001	0.028	−3.091; 3.180				
COVID-19_3	−3.159	−0.051	−1.255	−8.105; 1.787				

STAI Y1 = State-Trait Anxiety Inventory Form Y1; CI = confidence interval; COVID-19_1 = knowing of others who tested positive for COVID-19; COVID-19_2 = knowing of others who died of COVID-19; COVID-19_3 = having had contact with others who tested positive for COVID-19. * *p* < 0.05; ** *p* < 0.01.

**Table 3:** Hierarchical multiple regression predicting BDI-II scores from sociodemographic variables, health-related items, and COVID-19-related questions in the student group (*N* = 478).

BDI-II								
Predictors	B	*β*	*t*	95% CI	Adj R^2^	F	∆R^2^	∆F
**Model 1**					0.028	4.450 **	0.036	4.450 **
Age	0.084	0.025	0.480	−0.260; 0.428				
Gender	−3.317	−0.150	−3.290 **	−5.299; −1.336				
Marital status	0.710	0.015	0.335	−3.453; 4.873				
Educational level	−2.677	−0.144	−2.841 **	−4.529 −0.826				
**Model 2**					0.143	14.230 **	0.117	32.598 **
Age	−0.078	−0.023	−0.472	−0.404; 0.247				
Gender	−2.494	−0.113	−2.573 *	−4.399; −0.589				
Marital status	1.429	0.031	0.717	−2.485; 5.343				
Educational level	−2.253	−0.121	−2.542 *	−3.995; −0.511				
Health evaluation	−2.198	−0.343	−7.994 **	−2.739; −1.658				
Health concern	0.196	0.049	1.135	−0.143; 0.534				
**Model 3**					0.143	9.859 **	0.006	1.100
Age	−0.088	−0.026	−0.533	−0.414; 0.238				
Gender	−2.471	−0.112	−2.538 *	−4.385; −0.557				
Marital status	1.521	0.033	0.763	−2.398; 5.439				
Educational level	−2.287	−0.123	−2.579 *	−4.030; −0.544				
Health evaluation	−2.192	−0.342	−7.925 **	−2.736; −1.649				
Health concern	0.216	0.054	1.241	−0.126; 0.558				
COVID-19_1	0.622	0.034	0.715	−1.088; 2.332				
COVID-19_2	0.670	0.024	0.515	−1.888; 3.228				
COVID-19_3	−3.182	−0.067	−1.550	−7.216; 0.853				

BDI-II = Beck Depression Inventory; CI = confidence interval; COVID-19_1 = knowing of others who tested positive for COVID-19; COVID-19_2 = knowing of others who died of COVID-19; COVID-19_3 = having had contact with others who tested positive for COVID-19. * *p* < 0.05; ** *p* < 0.01.

## Data Availability

The data presented in this study are available on request from the corresponding author. The data are not publicly available due to privacy protection.

## Supplementary Materials

The following are available online at https://www.mdpi.com/1660-4601/18/5/2503/s1, Table S1: Hierarchical multiple regression predicting STAI Y1 scores from sociodemographic variables, health-related items, and COVID-19-related questions in the worker group (*N* = 478). Table S2: Hierarchical multiple regression predicting BDI-II scores from sociodemographic variables, health-related items, and COVID-19-related questions in the worker group (*N* = 478).
