# Supplementary material for: Psychological Distress among Italian University Students Compared to General Workers during the COVID-19 Pandemic
Source: Int J Environ Res Public Health. 2021 Mar 3;18(5):2503. doi: 10.3390/ijerph18052503 (PMC7967629; doi:10.3390/ijerph18052503)
Supplement: Supplementary file 1 [file ijerph-18-02503-s001.pdf]

International Journal of Environmental Research and Public Health

MDPI

Table S1. Hierarchical multiple regression predicting STAI Y1 scores from sociodemographic variables, health-related items, and COVID-19-related questions in the worker group (N = 478).

				STAI Y1				
**Predictors**	**B**	**β**	**t**	**95% CI**	**Adj R^2**	**F**	**ΔR^2**	**ΔF**
**Model 1**					0.059	8.420 **	0.066	8.420 **
Age	0.010	0.002	0.044	−0.431; 0.451				
Gender	−7.001	−0.244	−5.421 **	−9.539; −4.463				
Marital status	2.571	0.043	0.948	-2.761; 7.904				
Educational level	−2.568	−0.106	−2.129 *	−4.938; −0.198				
**Model 2**					0.242	26.377 **	0.185	58.215 **
Age	−0.158	−0.035	−0.779	−0.557; 0.241				
Gender	−4.412	−0.153	−3.720 **	−6.742; −2.081				
Marital status	3.028	0.050	1.242	−1.763; 7.819				
Educational level	−2.009	−0.083	−1.853	−4.140; 0.122				
Health evaluation	−2.273	−0.273	−6.763 **	−2.933; −1.612				
Health concern	1.766	0.341	8.382 **	1.352; 2.180				
**Model 3**					0.240	17.740 **	0. 003	0.601
Age	−0.166	−0.037	-0.817	−0.565; 0.233				
Gender	−4.286	−0.149	−3.592 **	−6.630; −1.941				
Marital status	3.200	0.053	1.309	-1.604; 8.004				
Educational level	−2.035	−0.084	−1.873	−4.169; 0.100				
Health evaluation	−2.231	−0.268	−6.589 **	−2.897; −1.566				
Health concern	1.792	0.346	8.417 **	1.373; 2.210				
COVID-19_1	−0.291	−0.012	−0.273	−2.386; 1.804				
COVID-19_2	0.045	0.001	0.028	−3.091; 3.180				
COVID-19_3	−3.159	−0.051	−1.255	−8.105; 1.787				

STAI Y1 = State-Trait Anxiety Inventory Form Y1; CI = Confidence Interval; COVID-19_1 = Knowing of others who are positive for COVID-19; COVID-19_2 = Knowing of others who died of COVID-19; COVID-19_3 = Having had contacts with others positive for COVID-19. * p<.05; ** p<.01.

Table S2. Hierarchical multiple regression predicting BDI-II scores from sociodemographic variables, health-related items, and COVID-19-related questions in the worker group (N = 478).

				BDI-II				
**Predictors**	**B**	**β**	**t**	**95% CI**	**Adj R^2**	**F**	**ΔR^2**	**ΔF**
**Model 1**					0.028	4.450 **	0.036	4.450 **
Age	0.084	0.025	0.480	−0.260; 0.428				
Gender	−3.317	−0.150	−3.290 **	−5.299; −1.336				
Marital status	0.710	0.015	0.335	−3.453; 4.873				
Educational level	−2.677	−0.144	−2.841**	−4.529; −0.826				
**Model 2**					0.143	14.230 **	0.117	32.598 **
Age	−0.078	−0.023	−0.472	−0.404; 0.247				
Gender	−2.494	-0.113	-2.573 *	-4.399; −0.589				
Marital status	1.429	0.031	0.717	−2.485; 5.343				
Educational level	−2.253	−0.121	−2.542 *	−3.995; −0.511				
Health evaluation	−2.198	−0.343	−7.994 **	−2.739; -1.658				

Health concern	0.196	0.049	1.135	−0.143; 0.534				
**Model 3**					0.143	9.859 **	0.006	1.100
Age	−0.088	−0.026	−0.533	−0.414; 0.238				
Gender	−2.471	−0.112	−2.538 *	-4.385; -0.557				
Marital status	1.521	0.033	0.763	−2.398; 5.439				
Educational level	−2.287	−0.123	−2.579 **	−4.030; −0.544				
Health evaluation	−2.192	−0.342	−7.925 **	−2.736; −1.649				
Health concern	0.216	0.054	1.241	−0.126; 0.558				
COVID-19_1	0.622	0.034	0.715	−1.088; 2.332				
COVID-19_2	0.670	0.024	0.515	−1.888; 3.228				
COVID-19_3	−3.182	−0.067	−1.550	−7.216; 0.853				

BDI-II = Beck Depression Inventory; CI = Confidence Interval; COVID-19_1 = Knowing of others who are positive for COVID-19; COVID-19_2 = Knowing of others who died of COVID-19; COVID-19_3 = Having had contacts with others positive for COVID-19. * p<.05; ** p<.01.
